# Hydrophobia, with Autopsy

**Published:** 1879-07

**Authors:** Henry M. Lyman


### ARTICLE VI.

*Hydrophobia, with Autopsy.*

Late in the evening of February 23, 1877, a man was brought in a cart to the Cook County Hospital. He was escorted by a policeman to whose care he had been intrusted, but before he could be carried to a bed, in the very doorway itself, he ceased to breathe. The policeman then produced a note from a physician in this city, certifying that the patient was bitten by a dog, December 12, 1876, and that he had been now for four days exhibiting all the symptoms of hydrophobia. The hospital clerk recorded these statements, and then proceeded to lose the note, as well as all memory of the name and address of its author. Policeman and waggoner in like manner disappeared, and nothing remained but a friendless corpse, without any history beyond what has been now set forth.

On the following day, about eighteen hours after death, the body was opened in my presence. The lungs were engorged with black blood. The right side of the heart was distended in a similar manner. The left side of the heart was quite empty. The superficial vessels of the entire brain (cerebrum and cerebellum) seemed ready to burst with black blood. Exposure of the centrum ovale showed conspicuous puncta over its whole area. The cortical portion of the hemispheres, and all the gray nuclei in every portion of the brain, were congested, and, especially in the medulla oblongata, exhibited a beautiful peach blossom color. The membranes of the spinal cord, and the choroid plexuses shared in the general increase of vascularity. There was, however, no visible increase in. the amount of the cerebro-spinal fluid. The other viscera were healthy. The entire brain and the cervical portion of the cord were placed at once in the hands of Dr. O. C. Oliver, Director of the Rush Medical College Histological Laboratory, who made several hundred sections from different portions of the specimen, but with only negative results. His methods of procedure were as follows:

"1. Examination of sections of the frozen tissues in iodine serum.

"2. Examination of sections made as above, and stained with carmine dissolved in ammonia.

"3. Examination of similar sections in glycerine and acetic acid, instead of iodine serum.

"4. Examination of sections hardened in alcohol and stained with boro-carmine, treated with alcohol to which one-third of its volume of No. 8 acetic acid had been added.

"Some of these sections were mounted in Canada balsam; others in glycerine.

"Sections of the cerebellum, carried through the corpus dentatum, and sections of the medulla oblongata, and of the Gasserian ganglion, were prepared in the same way, and were examined with powers varying from forty to four hundred and twenty diameters, without the discovery of any abnormal appearances.

"When the specimens were treated with acetic acid and glycerine (20 per cent. of No. 8), a magnifying power of four hundred and twenty diameters revealed a very peculiar appearance in the ganglion cells of the corpus dentatum, and in other cells. I at first mistook this for *granular degeneration* of the cell contents, but a comparison with the appearance presented by similar cells under different treatment proved it to be an artificial product of the acetic acid treatment."

It may be objected that this was not a case of hydrophobia. It is certainly a matter for regret that I did not have the patient under observation *ab initio;* but the statement of the policeman, the medical certificate which undoubtedly did exist, and the macroscopical appearances at the autopsy, all concur to confirm me in the belief that we really had to deal with the results of a genuine case of the disease.

HENRY M. LYMAN, M.D.

NOTE.—May 24, 1879. After a careful comparison of his specimens with similar preparations made under the direction of Prof. Charcôt, in Paris, Dr. Oliver finds no reason for changing any of the opinions above expressed.

